# Comparing Outcomes of Moyamoya Disease and Moyamoya Syndrome in a Real‐World Scenario: A Cohort Study

**DOI:** 10.1111/cns.70165

**Published:** 2024-12-09

**Authors:** Xiao‐Peng Wang, Jing‐Jie Li, Qian‐Nan Wang, Gan Gao, Dan Yu, Qian Zhang, Si‐Meng Liu, Min‐Jie Wang, Xiang‐Yang Bao, Lian Duan

**Affiliations:** ^1^ Senior Department of Neurosurgery The First Medical Center of Chinese PLA General Hospital Beijing China; ^2^ Medical School of Chinese PLA Beijing China; ^3^ Senior Department of Neurosurgery The Eighth Medical Center of Chinese PLA General Hospital Beijing China; ^4^ Senior Department of Neurosurgery The Fifth Medical Center of Chinese PLA General Hospital Beijing China

**Keywords:** moyamoya disease, moyamoya syndrome, multicenter, propensity‐score matching, real‐world study, revascularization

## Abstract

**Background:**

Moyamoya disease (MMD) and moyamoya syndrome (MMS) are rare cerebrovascular conditions with unclear distinctions in clinical presentation and prognosis.

**Aim:**

This study assessed potential differences between MMD and MMS patients using real‐world data on clinical manifestations, surgical outcomes, and stroke risk factors.

**Methods:**

This multicenter, retrospective cohort study examined patients with MMD or MMS treated at three tertiary academic hospitals in China, with a mean follow‐up of 11.2 ± 3.1 years. Clinical differences were compared between MMD and MMS, and postoperative cerebrovascular events were compared between patients who underwent surgery and those with conservative management. Primary outcomes were postoperative ischemic and hemorrhagic strokes. Risk factors were evaluated via multivariate Cox regression analysis.

**Results:**

Of the 2565 patients, 2349 had MMD and 216 had MMS. After 1:1 propensity‐score matching, no significant differences were observed between these two cohorts. Surgical patients had fewer cerebrovascular events than those who received conservative treatment (HR, 0.487; 95% CI, 0.334–0.711; *p* < 0.001). Preadmission modified Rankin scale scores > 2 (HR, 3.139; 95% CI, 1.254–7.857; *p* = 0.015) and periprocedural complications (HR, 8.666; 95% CI, 3.476–21.604; *p* < 0.001) were independent stroke risk factors in patients with MMD. Periprocedural complications (HR, 31.807; 95% CI, 10.916–92.684; *p* < 0.001) increased stroke risk in patients with MMS.

**Conclusions:**

This real‐world study revealed substantial clinical overlap between MMD and MMS. Both groups derived significant benefits from surgical revascularization, suggesting distinction may not be necessary to guide surgical management decisions. Optimizing preoperative status and preventing periprocedural complications may improve outcomes in these rare cerebrovascular conditions.

**Trial Registration:**

This study has been registered in the Chinese Clinical trial registry (registration number: ChiCTR2200064160)

## Graphical abstract

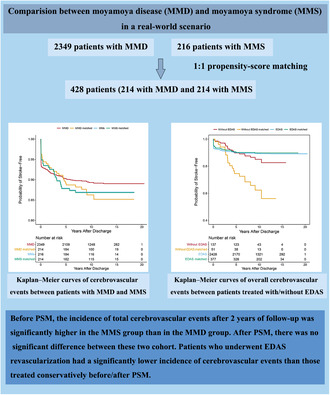

## 1 Introduction

Moyamoya disease (MMD) is characterized by chronic spontaneous stenosis and occlusion of the bilateral terminal portion of the internal carotid artery (ICA), occasionally involving the proximal middle and anterior cerebral arteries (MCA and ACA, respectively), accompanied by abnormal collateral vessel networks at the base of the brain [1, 2]. The term “moyamoya” refers to the characteristic angiographic findings of tiny vessels resembling a “puff of smoke” on digital subtraction angiography (DSA), regardless of the underlying etiology. When moyamoya patterns manifest with recognized underlying diseases such as atherosclerosis, autoimmune disease, neurofibromatosis type 1, or Down syndrome, they are classified as moyamoya syndrome (MMS). Both idiopathic MMD and MMS are described as moyamoya vasculopathies [2, 3]. Although the epidemiology of MMD has been well reported (the annual incidences in Japan, China, and North America are 1.13, 1.01, and 0.086 per 100,000, respectively), only one national survey has estimated the incidence of MMS (0.11 in Japan) [4, 5, 6]. Current research on the associations and distinctions between MMD and MMS is scarce and insufficient, particularly in large cohort studies [4, 5, 7, 8, 9, 10, 11, 12, 13]. Furthermore, the rarity of these conditions leads to uncertainty regarding their pathophysiological and prognostic similarities. Therefore, we conducted a comprehensive real‐world multicenter study with the largest sample size and longest follow‐up duration for MMD and MMS to date. This study aimed to investigate the similarities and differences between both conditions, assess their long‐term follow‐up outcomes to evaluate the benefit of surgical revascularization, and identify risk factors for recurrent cerebrovascular events. This could provide a better understanding of MMD and MMS and facilitate the development of customized treatment strategies.

## 2 Methods

### 2.1 Participants

**FIGURE 1:**
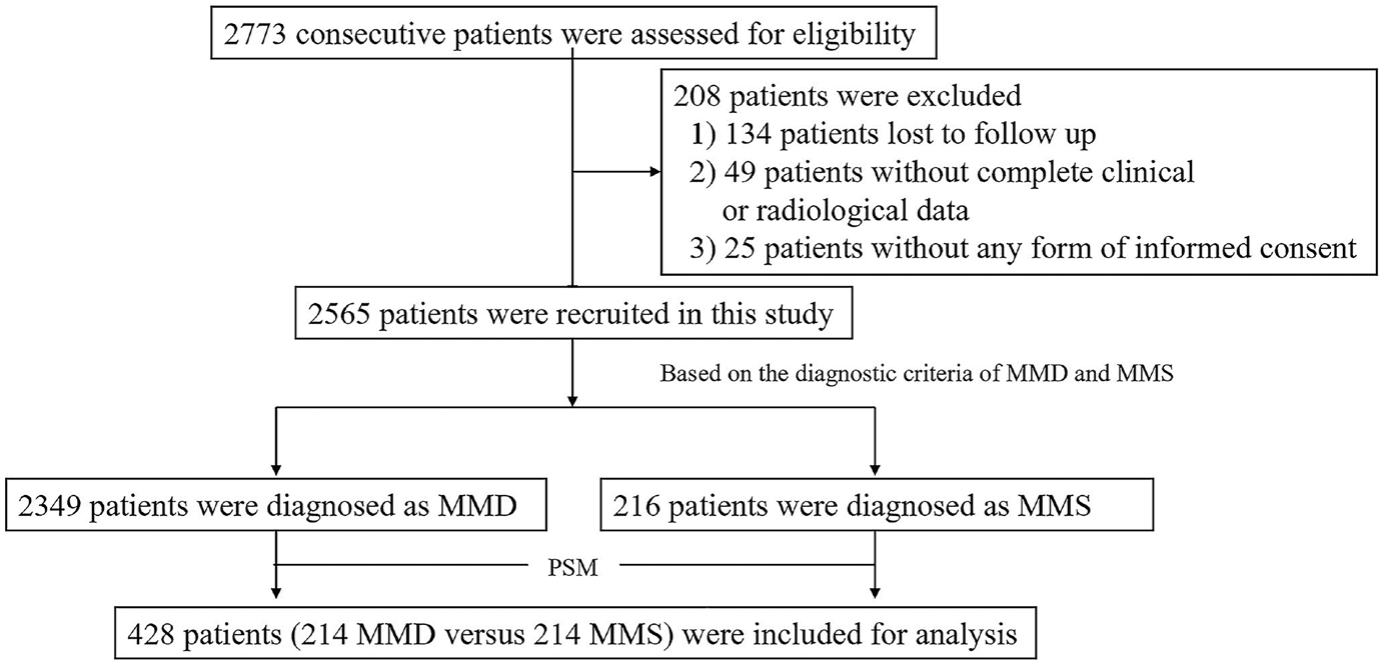
Study flowchart.

Consecutive patients with MMD and MMS were included retrospectively at three tertiary academic hospitals between November 2002 and July 2021. All patients underwent DSA examination and were diagnosed with MMD or MMS (details regarding underlying diseases are shown in Table S1) at three institutions according to the diagnostic criteria by the Research Committee on MMD (Spontaneous Occlusion of the circle of Willis) of the Ministry of Health, Labor Welfare, Japan [3]. The exclusion criteria were as follows: (1) insufficient imaging data, especially DSA data; (2) acute ischemic or hemorrhagic events within the 3 months before enrollment; (3) loss to clinical follow‐up; and (4) failure to provide informed consent. A total of 2565 patients (1605, 867, 93 patients from the Fifth Medical Center, the First Medical Center, and the Eighth Medical Center of Chinese PLA General Hospital, respectively) were included (Figure 1).

### 2.2 Clinical Data

A comprehensive assessment and collection of baseline demographic data of patients from all centers were conducted pre and posttreatment. This data included age, sex, stroke risk factors (hypertension, diabetes mellitus, hyperlipidemia), family history, modified Rankin scale (mRS) scores, treatment modalities, periprocedural complications (perioperative acute infarction, intracranial hemorrhage, subdural hematoma, epidural hematoma, subcutaneous effusion, transient ischemic attack [TIA], and incision infection), and recurrent cerebrovascular events (infarction or hemorrhage, excluding perioperative acute infarction and intracranial hemorrhage). Initial symptoms (identified by combining self‐reports with magnetic resonance imaging and computed tomography examinations) included headache, TIA, infarction, hemorrhage, and atypical symptoms; moreover, asymptomatic instances were also noted.

### 2.3 Radiological Assessment and Inter‐Rater Reliability

Patients were assessed using the Suzuki staging system, which evaluates the stenosis and occlusion of major cerebral vessels, along with fluctuations of moyamoya vessels observed through DSA [1]. Stenosed and moyamoya lesions within the posterior circulation artery were defined as posterior circulation involvement (PCI). Radiologic data were independently reviewed by an experienced neurosurgeon (XY‐B, 18 years of clinical experience) and an interventional neurologist (QN‐W, 10 years of clinical practice). The radiological data by the two raters were documented separately and interrater agreement was assessed using the weighted kappa test. To mitigate bias and ensure accurate and reliable results, the final results were decided by consensus.

### 2.4 Treatment Strategy and Follow‐Up Protocol

We primarily used encephaloduroarteriosynangiosis (EDAS) revascularization as our surgical approach owing to its high efficacy and safety [2, 3, 14, 15]. Surgical criteria for patients with MMD depend on the clinical symptoms, disease stage, and cerebral perfusion deficits affecting daily function, whereas patients with MMS require an additional criterion of refractory or worsening ischemia despite medical treatment of the underlying diseases. Considering the importance of functional brain areas in the anterior territories, symptoms, angiographic involvement, and perfusion condition, the initial surgery for moyamoya patients usually involves bilateral procedures in the MCA territory (typically in the dominant hemisphere). If there is PCI, posterior circulation surgery is performed 6 months after the anterior surgery, depending on the patient's overall state and tolerability.

Conservative treatment aims to manage symptoms and alleviate or eliminate episodes experienced by the patient. It is indicated for patients in one of the following categories: (1) those in the early stages of the disease or who experience mild intermittent symptoms that minimally impact daily living; (2) those in an advanced phase of the disease with high risk of perioperative cerebrovascular events who are concerned about operative risks and choose to decline surgical treatment voluntarily; (3) those with severe preoperative strokes and neurological deficits, making surgical treatment less meaningful; or (4) those with recent (within the past month) stroke or frequent recurrent strokes.

The follow‐up protocol for the study was as follows. All surgically and conservatively managed patients were enrolled in a standardized longitudinal follow‐up program documented in the hospital electronic medical record system from the three medical centers. Assessments were conducted via in‐person visits for hospitalized patients or annual remote video/telephone interactions for the others. Evaluations consisted of a neurological examination by a trained study physician, with administration of the mRS score to grade disability, as well as documentation of any recurrent cerebrovascular events (ischemic or hemorrhagic stroke).

### 2.5 Statistical Analysis

The baseline characteristics of the MMD and MMS groups were compared using the chi‐squared test (including Fisher's exact test) or Mann–Whitney *U* test. To minimize the impact of selection bias and confounding factors, we used propensity‐score matching (PSM) based on a 1:1 nearest‐neighbor matching algorithm with a caliper width of 0.2. The model incorporated the following predictor variables: sex, age, symptoms, family history, stroke risk factors, treatment modality (EDAS or not), preoperative mRS score, PCI, bilateral Suzuki stage, and unilateral lesion, with the evaluation of the propensity score based on a logistic regression model. Normality tests demonstrated that the age variable deviated significantly from a normal distribution. Moreover, given the differences in clinical phenotypes between pediatric and adult patients with moyamoya disease, we stratified this continuous variable into four clinically relevant age categories: 0–18, 19–36, 37–54, and ≥ 55 years. The Suzuki stage was classified into six groups (I–VI).

After PSM, primary outcomes were postoperative cerebrovascular events, including ischemic and hemorrhagic strokes. Univariate and multivariate Cox regression analysis and Kaplan–Meier survival analysis were used to investigate risk factors for cerebrovascular events in the MMD and MMS groups and in the groups with or without EDAS. The subgroup analyses of stroke risk factors were stratified by comorbidity burden, including “Hypertension, Diabetes mellitus, Hyperlipemia, Hypertension + Diabetes mellitus, Hypertension + Hyperlipemia, Diabetes mellitus + Hyperlipemia, Hypertension + Diabetes mellitus + Hyperlipemia, None (Hypertension or Diabetes mellitus or Hyperlipemia).” Statistical analysis was conducted using SPSS (version 25.0; IBM, Armonk, NY) and R (version 4.3.0; R Foundation for Statistical Computing, Vienna, Austria, https://www.r‐project.org/), with the R packages “forestploter,” “survminer,” and “survival.” Differences were considered statistically significant when the two‐tailed *p*‐values were < 0.05.

## 3 Results

### 3.1 Patient Characteristics

Of the 2565 included patients, 2349 (91.6%) had MMD and 216 (8.4%) had MMS (prevalence ratio: 0.09). Moreover, 208 patients were excluded based on the exclusion criteria: 134 patients were lost to follow‐up, 49 were without complete clinical or radiological data, and 25 were without any form of informed consent (Figure 1). Among the enrolled patients (age range: 1–77 years; 50.9% female; mean follow‐up duration: 11.2 ± 3.1 years, range 2.12–18.50 years with a median 10.26 years), 215 (8.4%) presented with unilateral lesions and 724 (28.2%) with PCI. Furthermore, 2428 (94.7%) patients underwent EDAS revascularization. A significant proportion of patients fell within the 37–54 years age range. The predominant presenting symptom was ischemic stroke. In terms of radiological severity, the majority of patients were classified as Suzuki stage IV or V. Of the 216 patients with MMS, one had a secondary diagnosis of Down syndrome, four had neurofibromatosis type 1, five had systemic lupus erythematosus, 45 had hyperthyroidism, and 159 had atherosclerosis. The initial comparison of baseline characteristics between the MMD and MMS groups before PSM revealed that patients with MMD were younger, had fewer stroke risk factors (hypertension, diabetes mellitus, hyperlipemia), had a lower Suzuki stage, and a higher propensity for hemorrhagic cerebrovascular events; however, excellent consistency was observed between the two matched groups after PSM (*n* = 214, respectively). Detailed characteristics before and after PSM are shown in Table 1

**TABLE 1:** Comparison of the baseline characteristics between patients of MMD and MMS.

Patient characteristics	Before PSM				After PSM			
	All cases (*n* = 2565)	MMD (*n* = 2349)	MMS (*n* = 216)	*p*‐value	All cases (*n* = 428)	MMD (*n* = 214)	MMS (*n* = 214)	*p*‐value
Age, years				0.000***				0.101
≤ 18	709 (27.6%)	695 (29.6%)	14 (6.5%)		42 (9.8%)	28 (13.1%)	14 (6.5%)	
19‐36	700 (27.3%)	655 (27.9%)	45 (20.8%)		81 (18.9%)	36 (16.8%)	45 (21.0%)	
37‐54	1020 (39.8%)	898 (38.2%)	122 (56.5%)		235 (54.9%)	113 (52.8%)	122 (57.0%)	
55‐72^d^	136 (5.3%)	101 (4.3%)	35 (16.2%)		70 (16.4%)	37 (17.3%)	33 (15.4%)	
Unilateral	215 (8.4%)	192 (8.2%)	23 (10.6%)	0.209	44 (10.3%)	23 (10.7%)	21 (9.8%)	0.874
Female	1306 (50.9%)	1189 (50.6%)	117 (54.2%)	0.318	234 (54.7%)	118 (55.1%)	116 54.2%)	0.923
PCI	724 (28.2%)	662 (28.2%)	62 (28.7%)	0.871	137 (32.0%)	76 (35.5%)	61 (28.5%)	0.147
Family history	141 (5.5%)	133 (5.7%)	8 (3.7%)	0.227	16 (3.7%)	8 (3.7%)	8 (3.7%)	1.000
Pre‐mRS > 2	203 (7.9%)	184 (7.8%)	19 (8.8%)	0.693	37 (8.6%)	19 (8.9%)	18 (8.4%)	1.000
Stroke risk factors								
Hypertension	436 (17.0%)	362 (15.4%)	74 (34.3%)	0.000***	140 (32.7%)	68 (31.8%)	72 (33.6%)	0.757
Diabetes mellitus	139 (5.4%)	108 (4.6%)	31 (14.4%)	0.000***	57 (13.3%)	27 (12.6%)	30 (14.0%)	0.776
Hyperlipidemia	229 (8.9%)	172 (7.3%)	57 (26.4%)	0.000***	103 (24.1%)	48 (22.4%)	55 (25.7%)	0.498
Initial symptom				0.000***				0.114^a^
Asymptomatic	27 (1.1%)	27 (1.1%)	0 (0)		4 (0.9%)	4 (1.9%)	0 (0)	
Headache	126 (4.9%)	115 (4.9%)	11 (5.1%)		29 (6.8%)	18 (8.4%)	11 (5.1%)	
TIA	1307 (51.0%)	1204 (51.3%)	103 (47.7%)		197 (46.0%)	96 (44.9%)	101 (47.2%)	
Infarction	538 (21.0%)	468 (19.9%)	70 (32.4%)		125 (29.2%)	55 (25.7%)	70 (32.7%)	
Hemorrhage	410 (16.0%)	390 (16.6%)	20 (9.3%)		48 (11.2%)	28 (13.1%)	20 (9.3%)	
Atypical	157 (6.1%)	145 (6.2%)	12 (5.6%)		25 (5.8%)	13 (6.1%)	12 (5.6%)	
Suzuki stage (bilateral, n*2)				0.000***				0.277^b^
0	215 (4.2%)	192 (4.1%)	23 (5.3%)		44 (5.1%)	23 (5.3%)	21 (4.9%)	
1	202 (3.9%)	188 (4.0%)	14 (3.2%)		27 (3.2%)	13 (3.0%)	14 (3.3%)	
2	619 (12.1%)	569 (12.1%)	50 (11.6%)		100 (11.7%)	50 (11.7%)	50 (11.7%)	
3	1000 (19.5%)	945 (20.1%)	55 (12.7%)		110 (12.9%)	55 (12.9%)	55 (12.9%)	
4	1608 (31.3%)	1480 (31.5%)	128 (29.6%)		269 (31.4%)	142 (33.2%)	127 (29.7%)	
5	1093 (21.3%)	988 (21.0%)	105 (24.3%)		214 (25.0%)	110 (25.7%)	104 (24.3%)	
6	393 (7.7%)	336 (7.2%)	57 (13.2%)		92 (10.7%)	35 (8.2%)	57 (13.3%)	

Abbreviations: MMD, moyamoya disease; MMS, moyamoya syndrome; PCI, posterior circulation involvement; PSM, propensity score matching; TIA, transient ischemic attack.

^a^ Using Fisher's exact test.

^b^ Using Mann–Whitney *U* test.

^c^ ***Significant *p*‐value (*p* < 0.001).

^d^ An additional 77‐year‐old patient was also included.

### 3.2 Long‐Term Outcomes Between MMD and MMS

**FIGURE 2:**
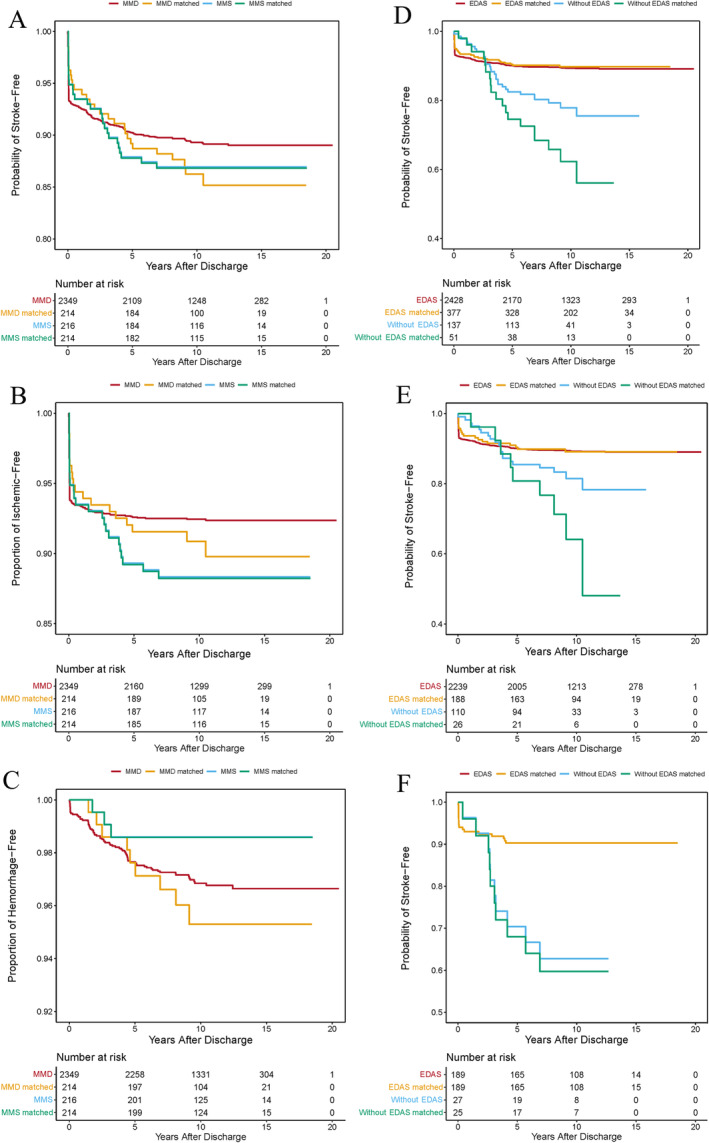
Kaplan–Meier curves of cerebrovascular events between patients with MMD and MMS for all cerebrovascular events (A), ischemic strokes (B), and hemorrhagic strokes (C). Kaplan–Meier curves of overall cerebrovascular events between patients treated with and without EDAS for all patients (D), patients with MMD (E), and patients with MMS (F). EDAS, encephaloduroarteriosynangiosis; MMD, moyamoya disease; MMS, moyamoya syndrome.

Among all patients, 289 (11.3%) had cerebrovascular events during follow‐up, including 209 (8.1%) ischemic and 80 (3.1%) hemorrhagic strokes (Table 2). Before PSM, the incidence of total cerebrovascular events (5.6% vs. 3.6%; hazard ratio [HR], 0.450; 95% confidence interval [CI], 0.242–0.836; *p* = 0.012) and ischemic stroke (4.6% vs. 0.6%; HR, 0.125; 95% CI, 0.056–0.282; *p* < 0.001) after 2 years of follow‐up was significantly higher in the MMS group than in the MMD group. No significant difference emerged in cerebrovascular event incidence across other follow‐up intervals. After PSM, 57 (13.3%) had cerebrovascular events during a mean follow‐up of 10.5 ± 3.0 years, including 45 (10.5%) with ischemic strokes and 12 (2.8%) with hemorrhagic cerebrovascular events. There was no significant difference between the two matched groups (Table 2, Figure 2A–C).

**TABLE 2:** Comparison of cerebrovascular events between patients with MMD and MMS during follow‐up.

	Before PSM				After PSM			
	All cases (*n* = 2565)	MMD (*n* = 2349)	MMS (*n* = 216)	*p*‐value	All cases (*n* = 428)	MMD (*n* = 214)	MMS (*n* = 214)	*p*‐value
Cerebrovascular events during overall follow‐up								
All events	289 (11.3%)	261 (11.1%)	28 (13.0%)	0.455	57 (13.3%)	29 (13.6%)	28 (13.1%)	0.891
Ischemic stroke	209 (8.1%)	184 (7.8%)	25 (11.6%)	0.072	45 (10.5%)	20 (9.3%)	25 (11.7%)	0.451
Hemorrhage	80 (3.1%)	77 (3.3%)	3 (1.4%)	0.148	12 (2.8%)	9 (4.2%)	3 (1.4%)	0.093
Cerebrovascular events in the first 2 years								
All events	217 (8.5%)	201 (8.6%)	16 (7.4%)	0.541	31 (7.2%)	15 (7.0%)	16 (7.5%)	0.847
Ischemic stroke	185 (7.2%)	170 (7.2%)	15 (6.9%)	0.843	29 (6.8%)	14 (6.5%)	15 (7.0%)	0.841
Hemorrhage	32 (1.2%)	31 (1.3%)	1 (0.5%)	0.278	2 (0.5%)	1 (0.5%)	1 (0.5%)	0.999
Cerebrovascular events after the first 2 years								
All events	72 (3.8%)	60 (3.6%)	12 (5.6%)	0.012*^a^	26 (6.1%)	14 (6.5%)	12 (5.6%)	0.680
Ischemic stroke	24 (0.9%)	14 (0.6%)	10 (4.6%)	0.000***^b^	16 (3.7%)	6 (2.8%)	10 (4.7%)	0.323
Hemorrhage	48 (1.9%)	46 (2.0%)	2 (0.9%)	0.299	10 (2.3%)	8 (3.7%)	2 (0.9%)	0.074

Abbreviations: MMD, moyamoya disease; MMS, moyamoya syndrome; PSM, propensity score matching.

^a^ *Significant *p*‐value (*p* < 0.05).

^b^ ***Significant *p*‐value (*p* < 0.001).

**FIGURE 3:**
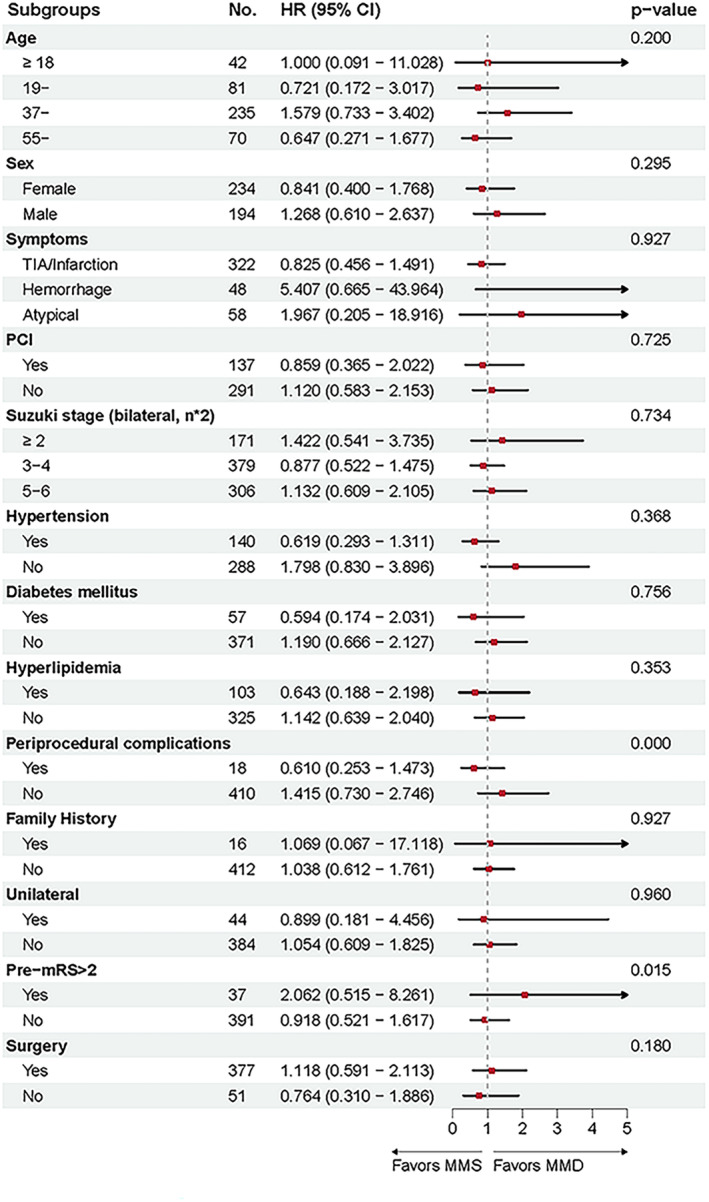
Subgroup analysis for comparison of prediction of cerebrovascular events between MMD versus MMS cohorts after matching. MMD, moyamoya disease; MMS, moyamoya syndrome; PCI, posterior circulation involvement; Pre‐mRS, pre‐admission modified Rankin Scale.

We conducted a subgroup analysis (Figure 3) of 13 subgroups to examine the relationship between MMD and MMS associated with total cerebrovascular events, revealing that MMS patients with periprocedural complications had a higher incidence of cerebrovascular events compared to the MMD patients (HR, 0.610; 95% CI, 0.253–1.473; *p* < 0.001). Furthermore, MMD patients with preoperative mRS scores > 2 had more cerebrovascular events compared to the MMS patients (HR, 2.062; 95% CI, 0.515–8.261; *p* = 0.015).

### 3.3 Surgical Benefit and Risk Factors of Cerebrovascular Events in MMD and MMS

Before PSM, 2239 (95.3%) and 189 (87.5%) patients underwent EDAS revascularization, and 110 (4.7%) and 27 (12.5%) patients received conservative treatment in the MMD and MMS groups, respectively. Patients who underwent EDAS revascularization had a significantly lower incidence of cerebrovascular events than those treated conservatively (HR, 0.487; 95% CI, 0.334–0.711; *p* < 0.001; Figure 2D), and this was consistent for both unmatched MMD (HR, 0.595; 95% CI, 0.377–0.939; *p* = 0.026; Figure 2E) and MMS (HR, 0.246; 95% CI, 0.113–0.533; *p* < 0.001; Figure 2F) groups. After PSM, patients treated with EDAS revascularization had a lower incidence of cerebrovascular events than those treated conservatively (HR, 0.256; 95% CI, 0.148–0.445; *p* < 0.001; Figure 2D). This difference was similar in the matched MMD (HR, 0.291; 95% CI, 0.132–0.641; *p* = 0.002; Figure 2E) and MMS (HR, 0.225; 95% CI, 0.104–0.489; *p* < 0.001; Figure 2F) groups. Furthermore, the neurological status of the patients improved significantly after treatment with EDAS revascularization in both the MMD and MMS groups (*p* = 0.004 and 0.003, respectively; Figure S1).

In the multivariate Cox regression model, all variables with *p* < 0.05 in the univariate analysis were incorporated. The results showed that EDAS (HR, 0.191; 95% CI, 0.077–0.471; *p* < 0.001) was a protective factor, whereas periprocedural complications (HR, 8.666; 95% CI, 3.476–21.604; *p* < 0.001) and preoperative mRS scores > 2 (HR, 3.139; 95% CI, 1.254–7.857; *p* = 0.015) were risk factors in the MMD group (Table S2). Additionally, EDAS (HR, 0.101; 95% CI, 0.031–0.330; *p* < 0.001) was a protective factor, whereas periprocedural complications (HR, 31.807; 95% CI, 10.916–92.684; *p* < 0.001) was a risk factor in the MMS group (Table S3).

### 3.4 Comparison Between Atherosclerosis and Hyperthyroidism

Among the 214 patients in the matched MMS group, atherosclerosis and hyperthyroidism were diagnosed in 159 patients and 45 patients, respectively. The baseline clinical characteristics of patients with atherosclerosis and hyperthyroidism are summarized in Table S4. Compared with the atherosclerosis group, the hyperthyroidism group showed a younger age (*p* < 0.001), more female patients (86.7% vs. 44.7%; *p* < 0.001), an earlier Suzuki stage (*p* = 0.048), and a lower incidence of hypertension (13.3% vs. 40.9%; *p* = 0.001), diabetes mellitus (4.4% vs. 17.0%; *p* = 0.031), and hyperlipidemia (4.4% vs. 33.3%; *p* < 0.001).

A total of 136 patients with atherosclerosis and 44 with hyperthyroidism underwent EDAS revascularization (mean follow‐up duration: 10.8 ± 3.1 years). Among them, 13 (9.6%) and 5 (11.4%) patients developed cerebrovascular events after EDAS treatment in the atherosclerosis and hyperthyroidism groups, respectively. There was no significant difference in the recurrence of cerebrovascular events during the short‐ and long‐term follow‐ups after surgical treatment between the atherosclerosis and hyperthyroidism groups (Table S5).

### 3.5 Radiological Interrater Reliability

Excellent intra‐interrater reliability was found for grading Suzuki stages bilaterally, with a weighted kappa of 0.943 (95% CI, 0.938–0.948; *p* < 0.001). Among 5130 hemispheres, there were only 471 inconsistencies between the two radiological raters. Final assessments were reached through duplicate review and consensus.

## 4 Discussion

### 4.1 Findings

This first large‐scale, real‐world PSM analysis provides novel insights into MMD and MMS by assessing long‐term outcomes from multiple centers. The key findings are as follows. First, both MMD and MMS demonstrated substantial clinical overlap with mild distinguishing features, even among MMS subtypes. Second, patients in MMD and MMS cohorts shared similarly favorable EDAS revascularization outcomes. Finally, perioperative complications were associated with increased recurrent cerebrovascular events in both groups. This study provides novel insights into the nosological controversy surrounding MMD and MMS and supports the personalized application of surgical revascularization for these rare angiopathies.

### 4.2 Demographic Characteristics

The current diagnostic criteria for MMD do not include an official definition of MMS. Various terms, including “quasi‐MMD” or “akin to MMD,” “rui‐MMD,” and “moyamoya phenomenon,” have been used in the literature [3, 16]. Although substantial progress has been made in clinical and fundamental research on MMD worldwide, the clinical characteristics and long‐term outcomes of patients with MMS remain unclear, particularly in large‐scale population‐based studies. A national epidemiological survey of MMS in Japan reported that the incidence of MMS is approximately one‐tenth of that observed in MMD, and we observed an analogous prevalence ratio [4].

Geographic variations exist, with a higher MMS prevalence within the moyamoya vasculopathy spectrum and differing subtype proportions between Western and East Asian countries [6, 17]. Neurofibromatosis type 1 and sickle cell disease are more frequently encountered in Western countries, whereas atherosclerosis and hyperthyroidism are predominant in East Asia. The age distribution of MMS in mainland China seems to differ from previous studies in other regions reporting two peaks [4, 6, 7, 10, 17, 18]. Our study showed a single peak at 45–49 years (Figure S2) without juvenile prominence, potentially due to a larger proportion of MMS patients being attributed to atherosclerosis. Another reason could be that children with associated diseases received pediatric care rather than neurosurgical interventions. Furthermore, a female predominance was not observed in our study (female/male ratio: 1.18:1), demonstrating a distinctive pattern observed exclusively among MMS patients in mainland China [8]. Previous studies have demonstrated a twofold higher prevalence of unilateral lesions in MMS than in MMD [8, 10]. However, our findings revealed a concordant proportion of unilateral lesions in MMS and MMD (approximately 10%), which is consistent with the prevalence observed in a Caucasian population [18]. The underlying reason for this lies in the systemic nature of the underlying diseases, which appears to preclude the occurrence of solely unilateral differences in lesions. Similar to previous research, our study revealed that ischemic symptoms (TIA and infarction) predominated as the primary clinical phenotypes in both MMD (71.2%) and MMS (80.1%), with a comparatively reduced occurrence of intracranial hemorrhage in MMS (9.3%) compared to MMD (16.6%). Family history and PCI were not significantly different between MMD and MMS, which is consistent with a previous epidemiological study on MMD in mainland China [19].

Among our MMS patients, concomitant atherosclerosis and hyperthyroidism were the prevailing subtypes. Patients with hyperthyroidism were younger, more often female, and had a lower incidence of hypertension, diabetes mellitus, and hyperlipidemia than those with atherosclerosis. This dissimilarity may be ascribed to the intrinsic epidemiological features of the underlying diseases [20, 21]. Moreover, patients with hyperthyroidism showed an earlier Suzuki stage compared to those with atherosclerosis, possibly due to their younger age and fewer vascular risk factors. Another contributing factor could be the favorable effects of appropriately elevated thyroid hormone levels on fewer vascular risk factors, consequently perturbing disease progression [22, 23]. Further studies are warranted to elucidate the mechanisms underlying the association between hyperthyroidism and the phenotypic presentation of MMS.

### 4.3 Stroke‐Free Survival and Risk Factors for Cerebrovascular Events

Our results demonstrate that patients with MMS have a higher incidence of long‐term ischemic cerebrovascular events than those with MMD, reflecting the higher rates of stroke risk burden in patients with MMS, which require precise and tailored pharmacological interventions for long‐term management. However, after rigorous adjustments for all clinical characteristics and stroke risk variables, no substantial differences were found in the occurrence of cerebrovascular events between the matched MMS and MMD groups, nor between the atherosclerosis and hyperthyroidism subgroups of MMS patients. Previous studies also showed comparable stroke‐free survival between these groups [7, 8, 9]. Nonetheless, a study conducted in the United States identified a higher incidence of stroke in patients with moyamoya disease (MMD) compared to those with moyamoya syndrome (MMS). This difference may be attributed to the underlying subtype heterogeneity within moyamoya syndrome. The study cohort predominantly consisted of individuals with sickle cell disease, neurofibromatosis type 1, and Down syndrome, characterized by a younger age and fewer vascular risk factors compared to East Asian groups where atherosclerotic and hyperthyroid subtypes are more prevalent. It is important to consider these population differences when interpreting prognostic comparisons between different geographic regions. There are presently no definitive therapies to halt vascular progression in moyamoya vasculopathy. The efficacy of pharmacological stroke prevention strategies including antiplatelet agents like acetylsalicylic acid and anticoagulants such as warfarin or heparin remain unknown [24, 25]. According to the guidelines for MMD in Japan and AHA/American Stroke Association, the use of antiplatelet medications such as aspirin in ischemic moyamoya patients may be considered reasonable for preventing further ischemic strokes and improving cognition [3, 12]. However, the potential role of aspirin in causing cerebral hemorrhagic events necessitates cautious use [3, 26, 27]. While bone marrow transplantation temporarily stabilizes moyamoya vasculopathy associated with sickle cell disease and along with antithyroid therapy in moyamoya vasculopathy with hyperthyroidism, their effectiveness appears limited with subsequent progression possible [28, 29]. Consequently, the long‐term natural course and prognosis are generally unfavorable for most patients with moyamoya vasculopathy, as disease advancement and health decline tend to be inevitable given the current limitations of pharmacotherapy.

Currently, there is a consensus on employing indirect revascularization surgery to treat pediatric patients with MMD. However, the optimal approach to surgical treatment for adult patients remains controversial [3, 30, 31, 32, 33]. Direct revascularization surgery undoubtedly provides immediate benefits by increasing cerebral blood flow, whereas indirect revascularization necessitates a lengthier period for extracranial collateral vessel development to compensate. However, indirect revascularization effectively mitigates perioperative complications, including hyperperfusion syndrome [34]. A recent multicenter study conducted in North America found no significant difference in short‐term and long‐term stroke incidence between the two surgical modalities for MMD [35]. Consequently, three institutions in this study employed the simpler and less perioperative complication‐prone indirect revascularization as the primary approach for surgical intervention in MMD. Notably, our study emphasized the crucial role of EDAS interventions, as patients who underwent EDAS treatment demonstrated markedly higher stroke‐free survival and significantly better neurological improvement than those who did not, regardless of the disease group. These findings substantiate the benefits of indirect revascularization in preventing recurrent cerebrovascular events in both cohorts despite the potential heterogeneity in the pathological processes of MMD and MMS.

In patients with MMD, a preoperative mRS score > 2 was a positive factor for cerebrovascular events. Mikami et al. [36] reported that a high mRS score was associated with an increased incidence of postoperative epilepsy in MMD. Consistent with prior studies, we also found that approximately half (18/38, 47.4%) of the cerebrovascular events occurred within 1 month after EDAS revascularization in the two matched groups [2, 37, 38]. Additionally, all 18 cerebrovascular events were ischemic strokes, which might be due to the unstable hemodynamics of ischemic MMD [39].

Our multivariate analysis also identified periprocedural complications as being independently associated with increased cerebrovascular events. Periprocedural complications are not rare in moyamoya vasculopathy and it remains unclear whether they can be attributed to the effects of the surgical intervention [2, 40]. Hayashi et al. [41] demonstrated that altered cerebral hemodynamics after revascularization may contribute to ischemic cerebrovascular events. Despite the association between perioperative complications and postoperative cerebrovascular events, surgical revascularization resulted in significantly improved stroke‐free survival compared to conservative management. Thus, surgical revascularization remains the preferred treatment for moyamoya vasculopathy. It is imperative to continuously optimize surgical techniques and deliver personalized perioperative care to mitigate periprocedural complications.

### 4.4 Limitations

This study had some limitations. First, the retrospective design may introduce selection bias regarding the treatment approach; prospective randomized controlled trials are needed to validate our findings. Second, we exclusively examined EDAS revascularization; comparative prognoses after alternative surgical techniques require further study. Third, the presence of incomplete postoperative pharmacotherapy details due to the diverse comorbidities and inconsistencies in subtype and temporal factors. Forth, the participation in this study was limited to medical centers within a single country and population demographic differences remain a potential limitation affecting generalizability. However, this real‐world study provides valuable longitudinal insights from a large sample of rare moyamoya patients despite the inherent constraints of retrospective analysis.

### 4.5 Conclusion

Our real‐world study demonstrated a substantial clinical overlap between MMD and MMS. Moreover, surgical revascularization provided significant benefits to both groups, suggesting that a distinction between MMD and MMS may not be a requisite for guiding surgical management. Additionally, an increased focus on preoperative assessment and vigilance during the perioperative to prevent surgical complications may help reduce the incidence of delayed postoperative cerebrovascular events.

## Author Contributions

**Xiao‐Peng Wang:** conceptualization, formal analysis, writing – original draft, writing – review and editing. **Jing‐Jie Li:** formal analysis, writing – original draft, writing – review and editing. **Qian‐Nan Wang:** methodology, data curation, writing – original draft, writing – review and editing. **Gan Gao:** data curation, writing – original draft. **Dan Yu, Qian Zhang:** investigation, data curation. **Si‐Meng Liu, Min‐Jie Wang:** data curation. **Xiang‐Yang Bao:** conceptualization, methodology, writing – original draft, writing – review and editing. **Lian Duan:** conceptualization, investigation, supervision, writing – original draft, writing – review and editing.

## Ethics Statement

This cohort study from three tertiary academic hospitals was approved by the Research Ethics Board of Chinese PLA General Hospital (KY‐2022‐9‐69‐1) and was conducted in accordance with the principles set forth in the Declaration of Helsinki (2013) for studies involving human participants.

## Consent

Informed consent for study participation was obtained from all patients or their immediate family members through written materials, letters, or telephone contact.

## Conflicts of Interest

The authors declare no conflicts of interest.

## Supporting information


Data S1.

## Data Availability

Dates are available upon reasonable request. The original data were recorded and stored in Science Data Bank and the electronic databases at the Chinese PLA General Hospital and are available upon reasonable request with the permission of the corresponding authors.
