# Supplementary material for: Comparing Outcomes of Moyamoya Disease and Moyamoya Syndrome in a Real‐World Scenario: A Cohort Study
Source: CNS Neurosci Ther. 2024 Dec 9;30(12):e70165. doi: 10.1111/cns.70165 (PMC11628736; doi:10.1111/cns.70165)

Supplementary Table S1. The underlying diseases associated with moyamoya syndrome.

Associated underlying diseases	Detail
Specific diseases or conditions	Atherosclerosis (n=159), Brain tumors, Wilms tumor, Leptospirosis, Radiation therapy, Oral contraceptive
Autoimmune diseases	Systemic lupus erythematosus (n=5), Antiphospholipid antibody syndrome, Periarteritis nodosa, and Sjogren syndrome, Kawasaki disease
Hereditary diseases	Neurofibromatosis type 1 (n=4), Down's syndrome (n=1), Noonan syndrome, Turner's syndrome, Alagille's syndrome, William's syndrome, Marfan's syndrome, Tuberous sclerosis
Hematological diseases	Sickle cell disease, Fanconi's anemia, Spherocytosis, Protein C deficiency, Type II plasminogen deficiency, Pyruvate kinase deficiency
Metabolic diseases	Hyperthyroidism (n=45), Diabetes mellitus-type IA, Primary oxalosis

Supplementary Table S2. Univariate and multivariate cox regression analyses of risk factors of MMD for cerebrovascular events.

Characteristics	Univariate cox regression analyses				Multivariate cox regression analyses			
	p-value	HR	95% CI		*p*-value	HR	95% CI	
Age	**0.043***	1.624	1.071	2.595	0.291	1.277	0.811	2.012
Sex	0.254	1.531	0.736	3.183				
Symptoms	0.522	1.114	0.801	1.548				
PCI	0.737	1.137	0.537	2.408				
Left-Suzuki stage	0.617	1.066	0.829	1.372				
Right-Suzuki stage	0.705	1.049	0.820	1.340				
HT	0.323	1.462	0.689	3.101				
DM	0.739	1.197	0.416	3.443				
HL	0.328	0.590	0.205	1.698				
HT+DM	0.955	0.960	0.228	4.041				
HT+HL	0.178	0.254	0.035	1.869				
DM+HL	0.918	0.900	0.122	6.620				
HT+DM+HL	0.997	0.000	0.000	-				
None (HT or DM or HL)	0.316	0.688	0.331	1.430				
EDAS	**0.002****	0.290	0.132	0.640	**0.000*****	0.191	0.077	0.471
Periprocedural complications	**0.000*****	6.045	2.657	13.753	**0.000*****	8.666	3.476	21.604
Family History	0.891	0.870	0.118	6.397				
Unilateral	0.943	0.957	0.290	3.162				
Pre-mRS >2	**0.032***	1.365	1.028	1.813	**0.015***	3.139	1.254	7.857

MMD: moyamoya disease; PCI: Posterior circulation involvement; HT: hypertension; DM: diabetes mellitus; HL: hyperlipemia; EDAS: encephaloduroarteriosynangiosis; Pre-mRS: pre-admission modified Rankin scale.

Supplementary Table S3. Univariate and multivariate cox regression analyses of risk factors of MMS for cerebrovascular events.

Characteristics	Univariate cox regression analyses				Multivariate cox regression analyses			
	p-value	HR	95% CI		*p*-value	HR	95% CI	
Age	**0.019***	1.942	1.117	3.375	0.341	1.427	0.687	2.964
Sex	0.963	1.018	0.484	2.139				
Symptoms	0.211	0.757	0.489	1.171				
PCI	0.326	1.473	0.680	3.192				
Left-Suzuki stage	0.532	1.088	0.835	1.418				
Right-Suzuki stage	0.500	1.084	0.858	1.368				
HT	**0.001****	3.927	1.812	8.510	0.586	1.428	0.396	5.152
DM	0.063	2.255	0.958	5.307				
HL	0.989	0.994	0.423	2.339				
HT+DM	0.004	3.707	1.501	9.153	0.571	1.330	0.497	3.562
HT+HL	0.385	1.536	0.584	4.040				
DM+HL	0.650	0.629	0.086	4.634				
HT+DM+HL	0.968	0.960	0.130	7.068				
None (HT or DM or HL)	**0.007***	0.311	0.132	0.731	0.880	0.900	0.231	3.508
EDAS	**0.000*****	0.225	0.104	0.488	**0.000*****	0.101	0.031	0.330
Periprocedural complications	**0.000*****	13.485	6.346	28.657	**0.000*****	31.807	10.916	92.684
Family History	0.939	0.925	0.126	6.810				
Unilateral	0.887	1.091	0.329	3.614				
Pre-mRS >2	0.337	1.174	0.846	1.628				

MMS: moyamoya syndrome; PCI: Posterior circulation involvement; HT: hypertension; DM: diabetes mellitus; HL: hyperlipemia; EDAS: encephaloduroarteriosynangiosis; Pre-mRS: pre-admission modified Rankin scale.

Supplementary Table S4. Comparison of the baseline characteristics between the atherosclerosis and hyperthyroidism groups.

Patient characteristics	atherosclerosis (n = 159)	hyperthyroidism (n = 45)	*p*-value
Age			**0.000*** **
≤18	0 (0)	9 (20.0%)	
19-	21 (13.2%)	24 (53.3%)	
37-	106 (66.7%)	12 (26.7%)	
55-	32 (20.1%)	0 (0)	
Sex (Female)	71 (44.7%)	39 (86.7%)	**0.000*** **
Symptoms			0.349
Asymptomatic	0 (0)	0 (0)	
Headache	8 (5.0%)	1 (2.2%)	
TIA	74 (46.5%)	24 (53.3%)	
Infarction	50 (31.4%)	17 (37.8%)	
Hemorrhage	18 (11.3%)	1 (2.2%)	
Atypical	9 (5.7%)	2 (4.4%)	
PCI	45 (28.3%)	13 (28.9%)	1.000
Suzuki stage (bilateral, n*2)			**0.048***
0	15 (4.7%)	4 (4.4%)	
1	10 (3.1%)	3 (3.3%)	
2	37 (11.6%)	8 (8.9%)	
3	34 (10.7%)	17 (18.9%)	
4	89 (28.0%)	35 (38.9%)	
5	86 (27.0%)	16 (17.8%)	
6	47 (14.8%)	7 (7.8%)	
Hypertension	65 (40.9%)	6 (13.3%)	**0.001****
Diabetes mellitus	27 (17.0%)	2 (4.4%)	**0.031***
Hyperlipidemia	53 (33.3%)	2 (4.4%)	**0.000*** **
Periprocedural complications	18 (11.3%)	4 (8.9%)	0.789

Family History	7 (12.5%)	0 (0)	0.352
Unilateral	15 (9.4%)	4 (8.9%)	1.000
Pre-mRS >2	15 (9.4%%)	1 (2.2%)	0.204

TIA: transient ischemic attack; PCI: Posterior circulation involvement. Pre-mRS: pre-admission modified Rankin scale.

Supplementary Table S5. Comparison of cerebrovascular events between patients with atherosclerosis and hyperthyroidism after revascularization.

	Before PSM				After PSM			
	All cases (n = 180)	atherosclerosis (n = 136)	hyperthyroidism (n = 44)	*p*-value	All cases (n = 180)	atherosclerosis (n = 136)	hyperthyroidism (n = 44)	*p*-value
Cerebrovascular events during overall follow-up								
All events	18 (10.0%)	13 (9.6%)	5 (11.4%)	0.747	18 (10.0%)	13 (9.6%)	5 (11.4%)	0.747
Ischemic stroke	17 (9.4%)	12 (8.8%)	5 (11.4%)	0.638	17 (9.4%)	12 (8.8%)	5 (11.4%)	0.638
Hemorrhage	1 (0.6%)	1 (0.7%)	0 (0.0%)	0.569	1 (0.6%)	1 (0.7%)	0 (0.0%)	0.569
Cerebrovascular events in the first 2 years								
All events	14 (7.7%)	10 (7.4%)	4 (9.1%)	0.741	14 (7.7%)	10 (7.4%)	4 (9.1%)	0.741
Ischemic stroke	13 (7.2%)	9 (6.6%)	4 (10.1%)	0.620	13 (7.2%)	9 (6.6%)	4 (10.1%)	0.620
Hemorrhage	1 (0.6%)	1 (0.7%)	0 (0.0%)	0.569	1 (0.6%)	1 (0.7%)	0 (0.0%)	0.569
Cerebrovascular events after the first 2 years								
All events	4 (2.2%)	3 (2.2%)	1 (2.3%)	0.949	4 (2.2%)	3 (2.2%)	1 (2.3%)	0.949
Ischemic stroke	4 (2.2%)	3 (2.2%)	1 (2.3%)	0.944	4 (2.2%)	3 (2.2%)	1 (2.3%)	0.944
Hemorrhage	0 (0.0%)	0 (0.0%)	0 (0.0%)	-	0 (0.0%)	0 (0.0%)	0 (0.0%)	-

PSM: propensity score matching

Supplementary Figure S1. Functional outcome (measured by mRS) before and after operation were stratified and compared between MMD and MMS. mRS: modified Rankin scale; MMD: moyamoya disease; MMS: moyamoya syndrome.

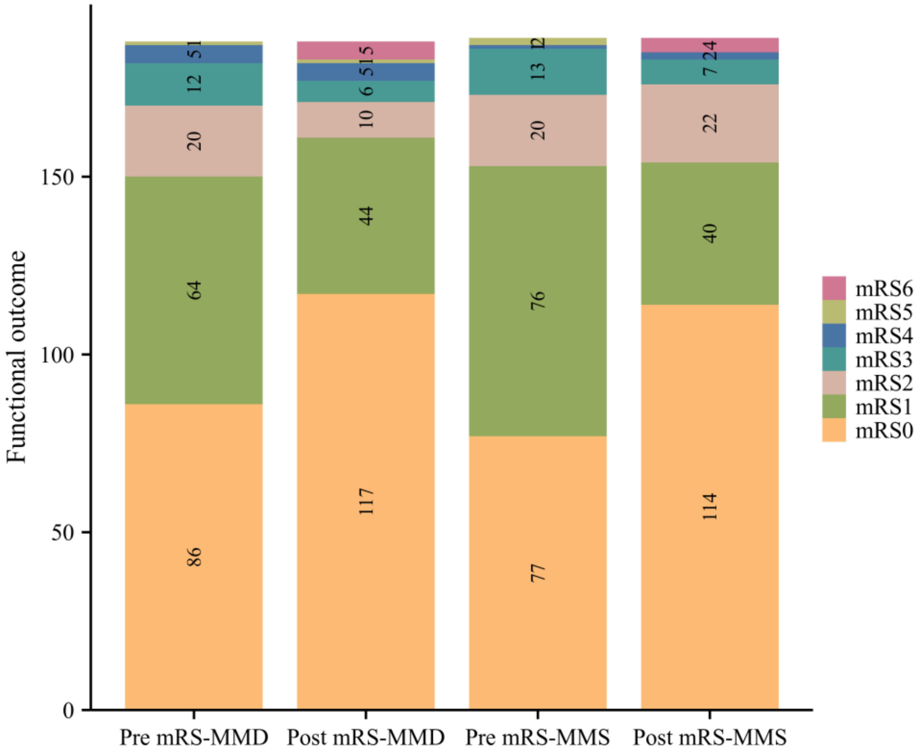

Supplementary Figure S2. Age distribution of MMD and MMS patients. MMD: moyamoya disease; MMS: moyamoya syndrome.

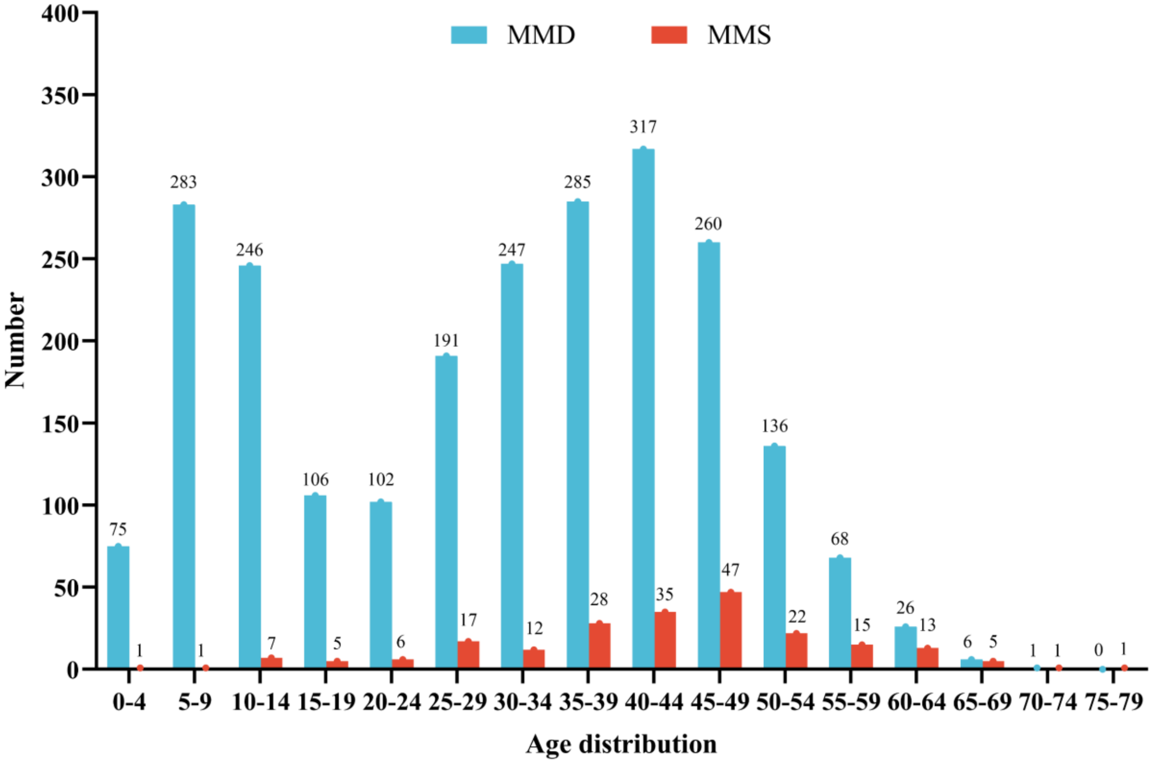

Supplement: Supplementary file 1 — Data S1. [file CNS-30-e70165-s001.pdf]
